# Obituary

**Published:** 1849-11

**Authors:** 


### ARTICLE III.

### OBITUARY.

DIED.---On the 7th Sept., of phthisis, Jno. Butterfield, M.D., Professor of the Principles and Practice of Medicine in the Starling Medical College of Columbus, Ohio, and one of the Editors of the Ohio Med. and Surg. Journal, one of the most able Medical Periodicals in the country. In the face of obstacles presented by a feeble constitution, Prof. Butterfield attained an honorable eminence in the Profession, and we deeply deplore his removal from among us, in the prime of his years and usefulness.

On the 18th August, at the early age of 31, Jno. E. McNairy, M.D., Superintendent of the Lunatic Asylum of the State of Tennessee.

